# Evaluating the safety and efficacy of SILS and SILS+1 port laparoscopic surgery for colorectal resection: a systematic review with meta-analysis and trial sequential analysis of RCTs

**DOI:** 10.3389/fonc.2025.1605040

**Published:** 2025-08-27

**Authors:** Kai Lu, Shilong Shu, Furui Zhong, Hua Yang, Yong Cheng, Faqiang Zhang

**Affiliations:** ^1^ Department of General Surgery, Zigong Fourth People’s Hospital, Zigong, Sichuan, China; ^2^ Department of Gastrointestinal Surgery, The First Affiliated Hospital of Chongqing Medical University, Chongqing, China

**Keywords:** reduced-port laparoscopic surgery, conventional laparoscopic surgery, colorectal cancer, colorectal resection, RLS

## Abstract

**Background:**

As minimally invasive techniques evolve, reduced-port laparoscopic colorectal resection, including single-incision (SILS) and single-incision plus one (SILS+1) approaches, has gained increasing clinical traction. However, whether it offers definitive advantages over conventional multiport laparoscopic surgery remains contentious. This meta-analysis aimed to comprehensively evaluate the safety and efficacy of reduced-port laparoscopic surgery (RLS) for colorectal resection and validate the robustness of findings through trial sequential analysis (TSA).

**Methods:**

A systematic literature search was conducted across Web of Science, PubMed, Cochrane Library, and Embase from database inception to March 21, 2025, to identify RCTs comparing RLS with conventional laparoscopic surgery (CLS) for colorectal resection. Methodological quality was assessed using the Cochrane Risk of Bias Tool. Meta-analyses were performed in RevMan 5.3, with TSA employed to control for random errors. Primary endpoints included operative time, intraoperative blood loss, intraoperative complications, postoperative complications, and postoperative pain.

**Results:**

Fourteen RCTs involving 1,713 patients were analyzed. Pooled data demonstrated no statistically significant differences between RLS and CLS in operative time (SMD: 0.29; 95% CI: −0.07 to 0.64; *p* = 0.11), intraoperative blood loss (SMD: 0.04; 95% CI: −0.06 to 0.15; *p* = 0.40), intraoperative complications (OR: 1.6; 95% CI: 0.88 to 2.88; *p* = 0.12), or postoperative complications (OR: 0.88; 95% CI: 0.67 to 1.17; *p* = 0.38). RLS was associated with significantly shorter incision length (SMD: −1.60; 95% CI: −2.37 to −0.83; *p* < 0.0001). Secondary outcomes, including complication severity, resection margins, lymph node harvest, time to first flatus, hospital stay, conversion rates, and anastomotic leakage, showed comparable results between approaches.

**Conclusion:**

RLS demonstrates comparable safety profiles and operative efficiency to conventional laparoscopic resection, and with its principal advantage being reduced incision length, this approach can serve as an alternative surgical option for patients.

## Background

In 2022, approximately 20 million new cancer cases were diagnosed globally, with colorectal cancer (CRC) accounting for 9.6% of total cases and ranking as the second leading cause of cancer-related mortality (1). Laparoscopic colorectal resection remains the gold standard for CRC treatment. The evolution of minimally invasive techniques has driven the development of reduced-port laparoscopic approaches, including single-incision (SILS) and single-incision plus one (SILS+1) port laparoscopic surgery. While CLS is associated with potential postoperative complications linked to multiple incisions, RLS offers advantages such as shorter incision length, enhanced cosmesis, and potentially reduced postoperative pain (2). Pioneering work by Remzi et al. (3) first demonstrated the feasibility of single-incision laparoscopic colorectal resection. Subsequently, Huscher et al. (4) provided Level I evidence through RCT, confirming comparable short-term safety profiles between SILS and conventional approaches. Recent investigations by Zhang X et al. (5) extended these findings, reporting equivalent 3-year disease-free survival (DFS) and 5-year overall survival (OS) for SILS+1 versus conventional laparoscopy in sigmoid and upper rectal cancer resection, alongside superior cosmetic outcomes. However, the purported short-term benefits of RLS remain controversial. Meta-analyses by ElSherbiny M et al. (6) identified higher conversion rates to open surgery with SILS, while Li FH et al. (7) reported increased intraoperative complications without comprehensive advantages. To address these conflicting findings, this meta-analysis incorporates recently published RCTs (up to 2024) evaluating both SILS and SILS+1 techniques, aiming to provide an updated synthesis of evidence on the safety and efficacy of reduced-port laparoscopic colorectal resection.

## Methods

This meta-analysis was conducted in accordance with the Preferred Reporting Items for Systematic Reviews and Meta-Analyses (PRISMA) guidelines (8) and prospectively registered with the International Prospective Register of Systematic Reviews (PROSPERO; Registration ID: CRD420251024445).

### Literature search strategy

Two independent researchers (S.L.S and F.R.Z) systematically searched Web of Science, PubMed, Cochrane Library, and Embase from inception to March 21, 2025. The Boolean logic-based strategy combined: Disease terms:(“Colorectal Neoplasms”[Mesh]) or (“colorectal tumor*” or “colorectal cancer*” or “colorectal carcinoma*”); Intervention terms:(“Laparoscopy”[Mesh]) or (“peritoneoscopy” or “celioscopy” or “laparoscopic assisted surgery”); Technique-specific terms:(“single-port” or “single-incision” or “reduced-port” or “SILS” or “SILS+1”). Search components were linked by and operators to refine results. No language filters were applied during initial screening.

### Eligibility criteria

#### Inclusion criteria

Population: Adults (≥18 years) with histologically confirmed colorectal adenocarcinoma. Intervention: Reduced-port Laparoscopic Surgery (SILS or SILS+1). Comparator: Conventional multiport laparoscopy (≥3 ports). Outcomes: Quantitative reporting of ≥1 predefined safety/efficacy parameter. Study design: Peer-reviewed randomized controlled trials (RCTs).

#### Exclusion criteria

Non-comparative designs (case reports, single-arm studies); Non-RCT publications (letters, reviews, conference abstracts); Non-English publications without validated translation; Animal or cadaveric studies; Robotic-assisted single-port procedures**;** Insufficient outcome data for quantitative synthesis.

### Risk of bias assessment

The methodological quality of the included randomized controlled trials (RCTs) was rigorously evaluated using the Cochrane Risk of Bias Tool. Two independent investigators (S.L.S. and F.R.Z.) assessed six critical domains of potential bias: selection bias (encompassing random sequence generation and allocation concealment), performance bias (blinding of participants and personnel), detection bias (blinding of outcome assessors), attrition bias (incomplete outcome data), reporting bias (selective outcome reporting), and other biases. Any discrepancies in assessment were resolved through consensus discussions or adjudication by a third senior researcher (H.Y.). Final judgments for each domain were categorized as “low risk,” “unclear,” or “high risk” to ensure standardized interpretation of study quality. This process adhered to the latest Cochrane guidelines (RoB 2.0 framework) to maintain methodological consistency across evaluations.

### Data extraction

All retrieved citations were imported into Zotero reference management software (Version 6.0) for systematic deduplication and screening. Two investigators (S.L.S. and F.R.Z.) independently performed a three-stage selection process: i) initial title/abstract screening, ii) full-text eligibility assessment, and iii) final inclusion confirmation. Discrepancies resolved through third-party arbitration (H.Y.). Standardized extraction templates were utilized to collect: i) Study identification: First author, publication year, country; ii) Demographic characteristics: Patient sex distribution, age (years), BMI (kg/m²). iii) Oncological parameters: Tumor location. iv) Intervention details: Reduced-port technique (SILS and SILS+1); v) Outcome metrics: Sample size per arm.

### Outcomes

Primary Outcomes: Operative time, intraoperative blood loss, intraoperative complications, postoperative complications. Secondary Outcomes: Surgical complication grades, proximal and distal resection margins, number of lymph nodes harvested, time to first postoperative flatus, length of hospital stay, incision length, conversion to open surgery, anastomotic leakage, postoperative pain. Postoperative complications were defined as any adverse events occurring within 30 days following the surgical procedure.

### Statistical analysis

All analyses were conducted using Review Manager (RevMan) version 5.3.1 (Copenhagen: The Nordic Cochrane Centre, The Cochrane Collaboration) (9). Continuous outcomes with heterogeneous measurement units were analyzed via standardized mean difference (SMD), while dichotomous outcomes were expressed as odds ratio (OR), both with 95% confidence interval (CI). Forest plots were generated for all outcomes. Heterogeneity was quantified using Higgins I² statistics, with I² >50% indicating substantial heterogeneity. A random-effects model was applied for I² >50%, and a fixed-effects model for I² ≤50%. Sensitivity analyses were performed for outcomes with significant heterogeneity (I² >50%). Subgroup analyses stratified postoperative complications by severity (Grade I-III). Funnel plots were constructed for primary outcomes with low heterogeneity (I² <50%) to assess publication bias.

### Trial sequential analysis

Trial sequential analysis (TSA) was conducted using TSA software version 0.9.5.10 (10) from the Copenhagen Trial Unit to control random errors and estimate the required information size. Continuous outcomes including operative time and intraoperative blood loss were analyzed by calculating the standardized mean difference and variance between RLS and CLS. For dichotomous outcomes such as intraoperative and postoperative complications, incidence rates were derived from the largest included randomized controlled trial. Key parameters included a two-sided type I error rate of α = 0.05 and 80% statistical power. The required information size was calculated based on the event rates、SMD and variance in the intervention and control groups. Final interpretations adhered to predefined monitoring boundaries: futility thresholds indicating sufficient evidence for equivalence, required information size achievement suggesting definitive conclusions, or boundary crossing supporting superiority or noninferiority.

### Ethical statement

As a meta-analysis of published RCTs, this study utilized existing aggregated data. All included trials had received ethics committee approval. No ethical review was required since no new human/animal subjects were involved.

## Results

### Study selection

**Figure 1:**
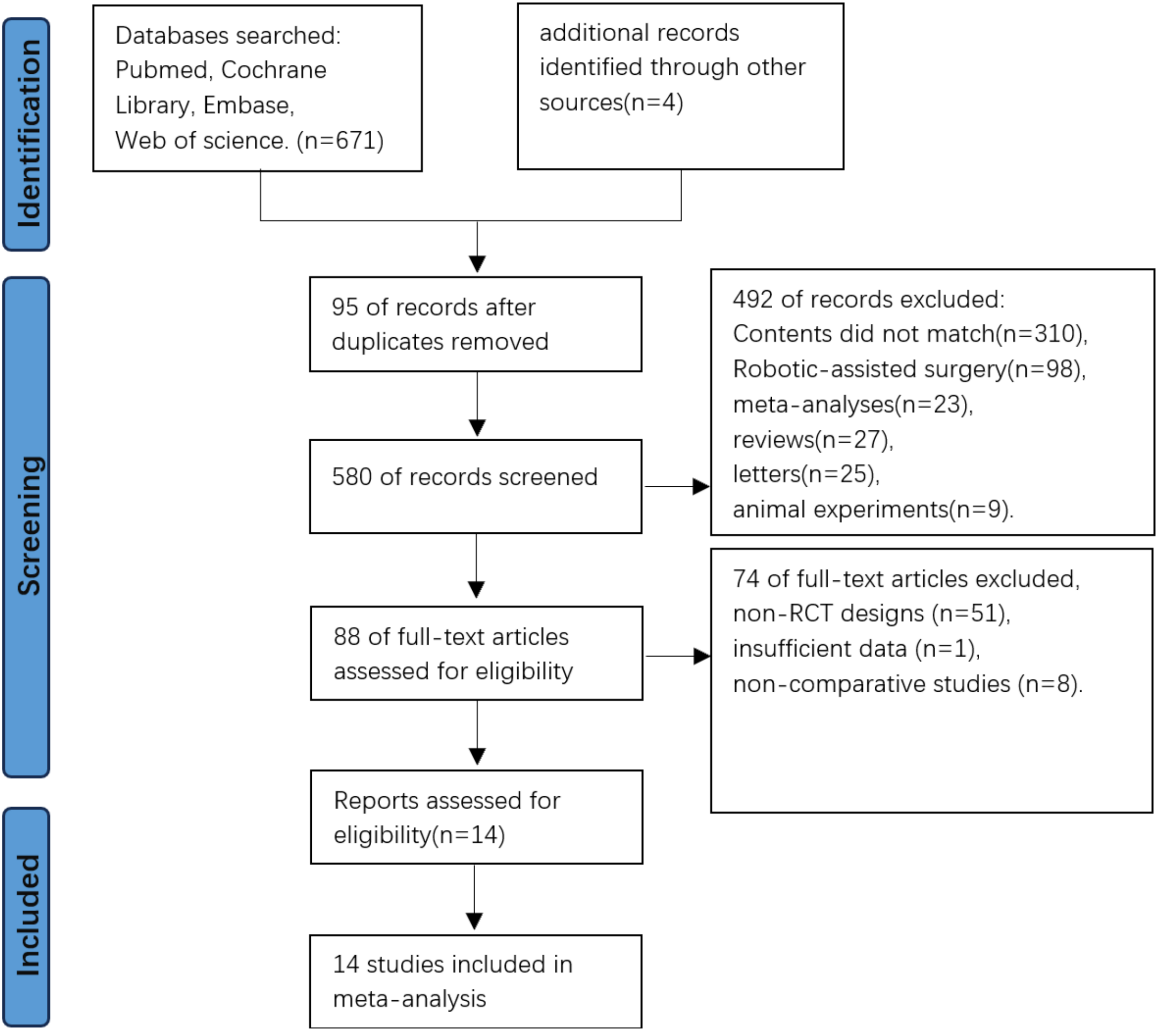
Flowchart of research screening process.

The initial database search yielded 671 records, supplemented by 4 additional records from other sources. After excluding 95 duplicates through Zotero 6.0 reference management software, 580 studies underwent title/abstract screening. Irrelevant studies (n=492) were excluded based on predefined criteria, leaving 88 articles for full-text evaluation. Following detailed assessment, 74 articles were excluded due to non-RCT designs, insufficient data, or non-comparative studies. Fourteen RCTs met all eligibility criteria and were included for meta-analysis and trial sequential analysis (**Figure 1**).

### Study characteristics

Fourteen randomized controlled trials (2, 4, 5, 11–21) involving a total of 1,713 patients were included in this meta-analysis, with 858 patients undergoing RLS and 855 patients undergoing CLS. Two studies required data consolidation: Watanabe et al. (12) published duplicate datasets in 2016 and 2021, which were merged for analysis, while partial overlapping data from Zhang X et al. (5) and Wang Y et al. (7) were pooled to avoid redundancy. Among the included studies, 12 articles reported outcomes for operative time and postoperative complications, revealing complication rates of 15.27% (117/766) in the RLS group and 16.93% (129/762) in the CLS group. Intraoperative blood loss data were analyzed in 11 studies, and intraoperative complications were evaluated in 5 studies, demonstrating occurrence rates of 5.89% (29/492) in the RLS group versus 3.69% (18/488) in the CLS group. A summary of baseline characteristics is presented in **Table 1**.

**Table 1:** Baseline information for inclusion in the study.

Author	Year	Surgical approach	Gender (M/F)		Age		BMI		Sample		Tumor location
			RLS	CLS	RLS	CLS	RLS	CLS	RLS	CLS	
Zhang X (5)	2023	SILS +1	49/43	56/37	56.9 ±11.5	57.2±11.7	22.8±2.7	23.0±3.1	92	93	Sigmoid colon Rectosigmoid rectum
Tang J (21)	2023	SILS	15/11	12/13	60.19±2.8	61.16±3.48	25.42±2.89	24.12±3.34	26	25	Rectum
Song Z (2)	2021	SILS	56/41	54/42	63 (54.5-69)	65 (56–70)	23.0 (2.8)	23.6 (3.2)	97	96	Right colon Left colon/Sigmoid colon/ rectum
Watanabe J (13, 20)	2016 2021	SILS	56/44	56/44	66.7 (8.8)	66.6 (8.9)	23.1 (3.32)	23.2 (3.32)	100	100	Ascending colon/ Sigmoid colon/ Rectosigmoid
Lee YS (19)	2021	SILS	97/82	99/81	63.4 (34–84)	62.6 (28–85)	24.3 (17.0–32.0)	24.3 (18.0–35.0)	179	180	Right colon/Left colon
Wang Y (18)	2019	SILS +1	49/43	56/37	56.9±11.5	57.2±11.7	22.8±2.7	23.0±3.1	92	93	Sigmoid colon/ Rectosigmoid/Rectum
Borowski DW (17)	2019	SILS	11/14	14/11	62 (19-83)	62.5 (26-83)	28 (20-45)	26 (19-51)	25	25	Right colon/ Left colon/Rectum
Maggiori L (16)	2018	SILS	29/33	28/35	48±17	51±18	23.7±3	23.8±4	62	63	Right colon/Left colon
Kang BM (15)	2018	SILS	50/43	51/37	62.4 (34–82)	62.3 (38–85)	24.4 (17.1–32.4)	24.2 (17.6–34.1)	93	88	Right colon /Left colon
Kang BM (14)	2017	SILS	19/12	16/15	63.2±11.4	62.2±9.4	24.0±3.0	24.5±3.0	31	31	Right colon/Left colon
Bulut O (12)	2015	SILS	8/12	8/12	69 (50–86)	73 (50-84)	24 (16–32)	24 (19–29)	20	20	Rectum
Poon JT (11)	2012	SILS	14/11	18/7	67 (37–83)	67 (57–81)	23.2 (16.9–28.8)	23.6 (16.5–28.2)	25	25	Right colon/Left colon/Sigmoid colon/rectum
Huscher CG (4)	2012	SILS	6/10	9/7	70±11	70±13	/	/	16	16	Right colon/Leftcolon

M, Male; F, Female; RLS, Reduced-port laparoscopic surgery; CLS, conventional laparoscopy surgery; SILS, Single-incision laparoscopy surgery.

### Quality assessment of included studies

**Figure 2:**
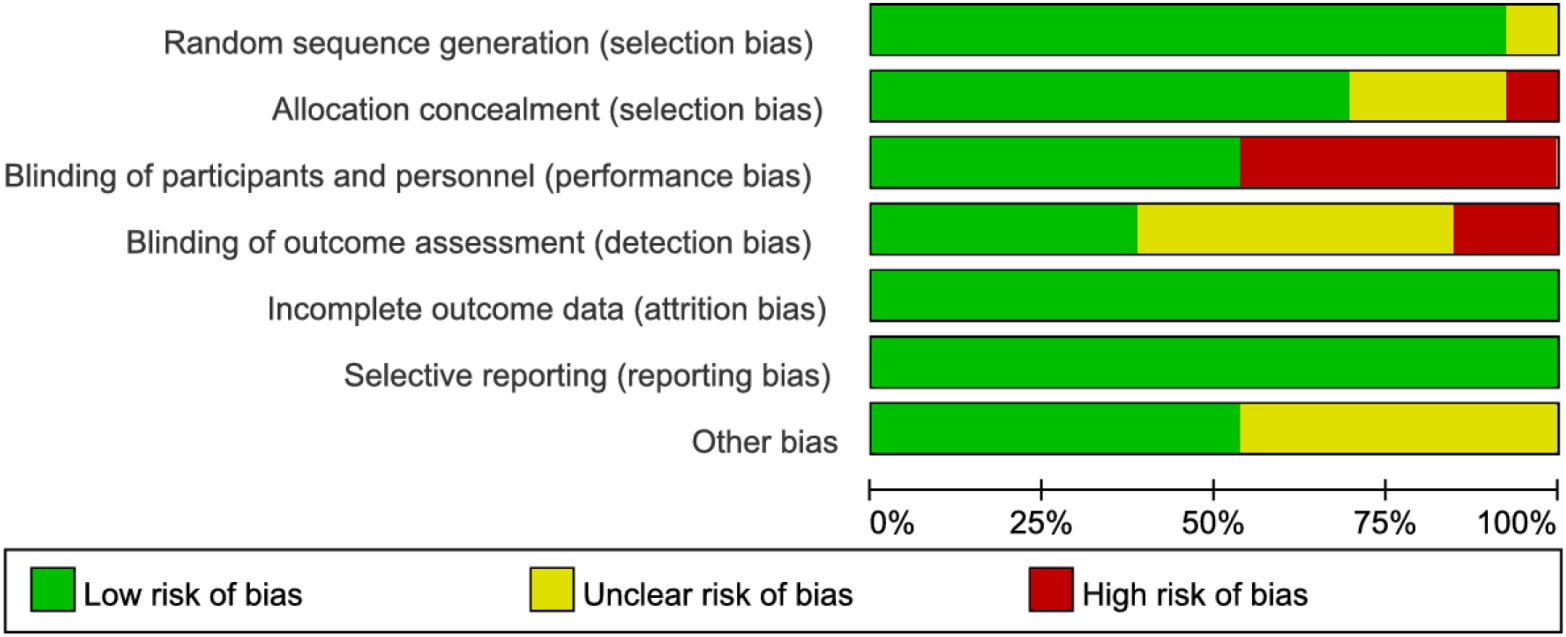
Risk of bias graph across included studies.

**Figure 3:**
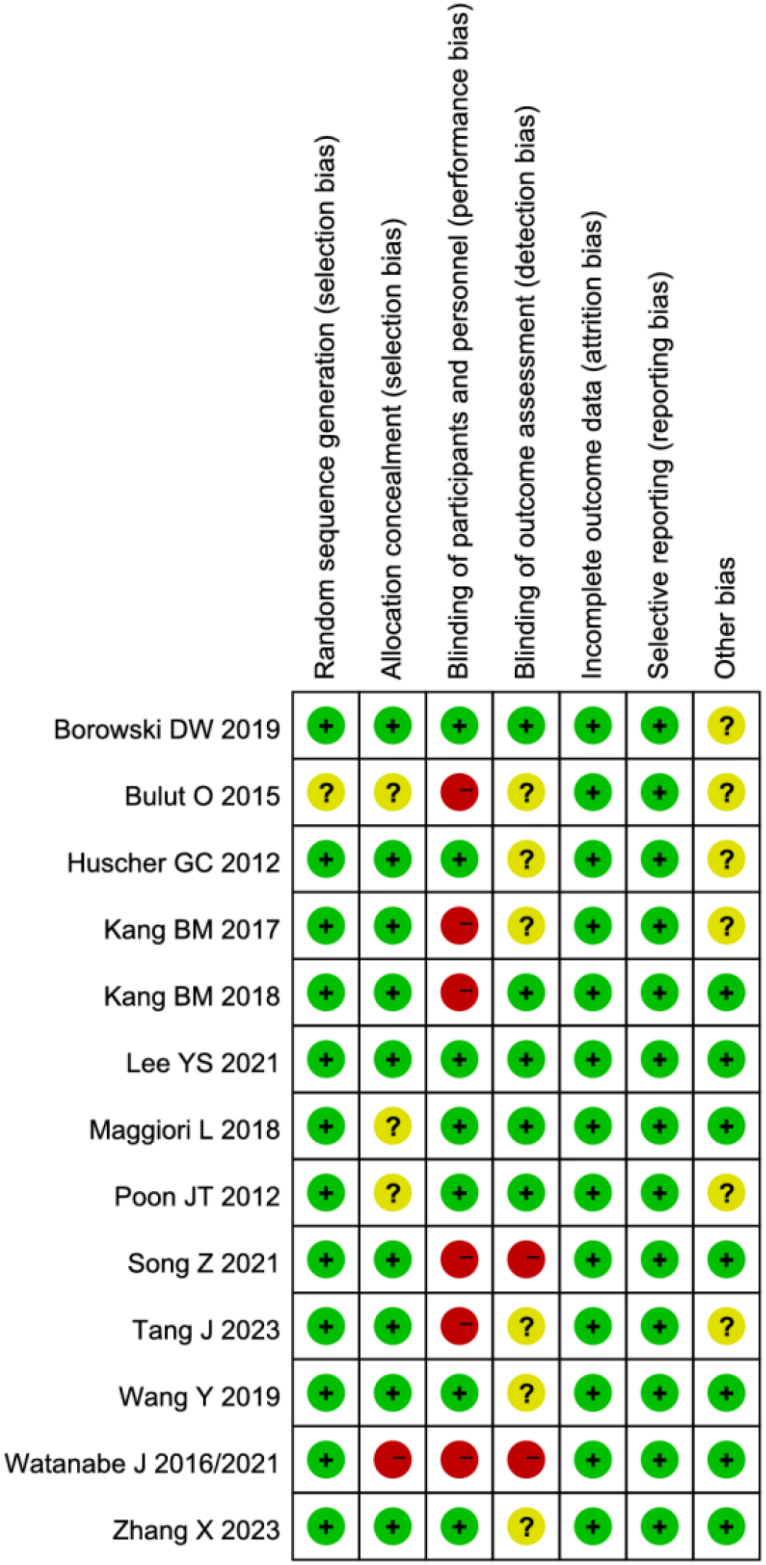
Risk of bias summary across included studies.

The methodological quality of the included studies was assessed using the Cochrane Risk of Bias tool. Notably, studies by Watanabe J et al. (13, 21) demonstrated high risk of selection bias due to inadequate allocation concealment. Seven studies (2, 12–15, 20, 21) exhibited high risk of performance bias as they failed to implement blinding of investigators and participants. Three studies (2, 13, 20) showed high risk of detection bias owing to lack of blinding in outcome assessment. While some studies were limited by relatively small sample sizes, the remaining articles demonstrated no high-risk biases. Overall, the methodological quality of the included studies was considered satisfactory (**Figures 2**, **3**).

### Primary outcomes

#### Operative time and intraoperative blood loss

**Figure 4:**
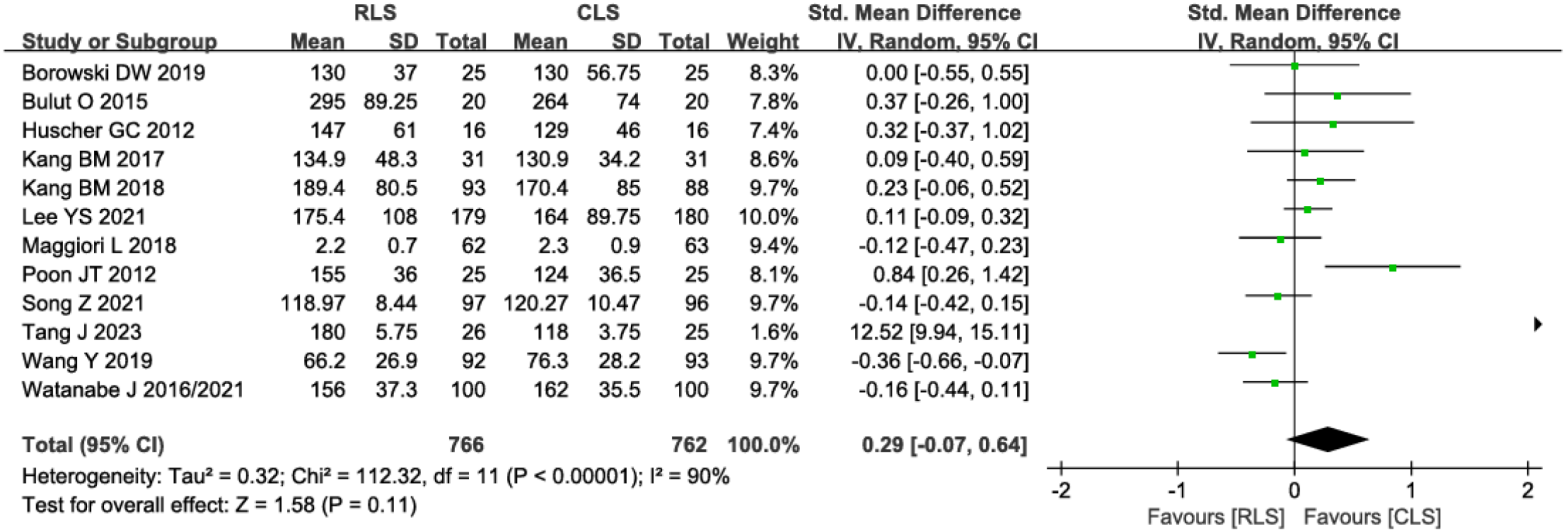
Forest plot of operative time comparing RLS and CLS. RLS, reduced-port laparoscopic surgery; CLS, conventional laparoscopic surgery.

**Figure 5:**
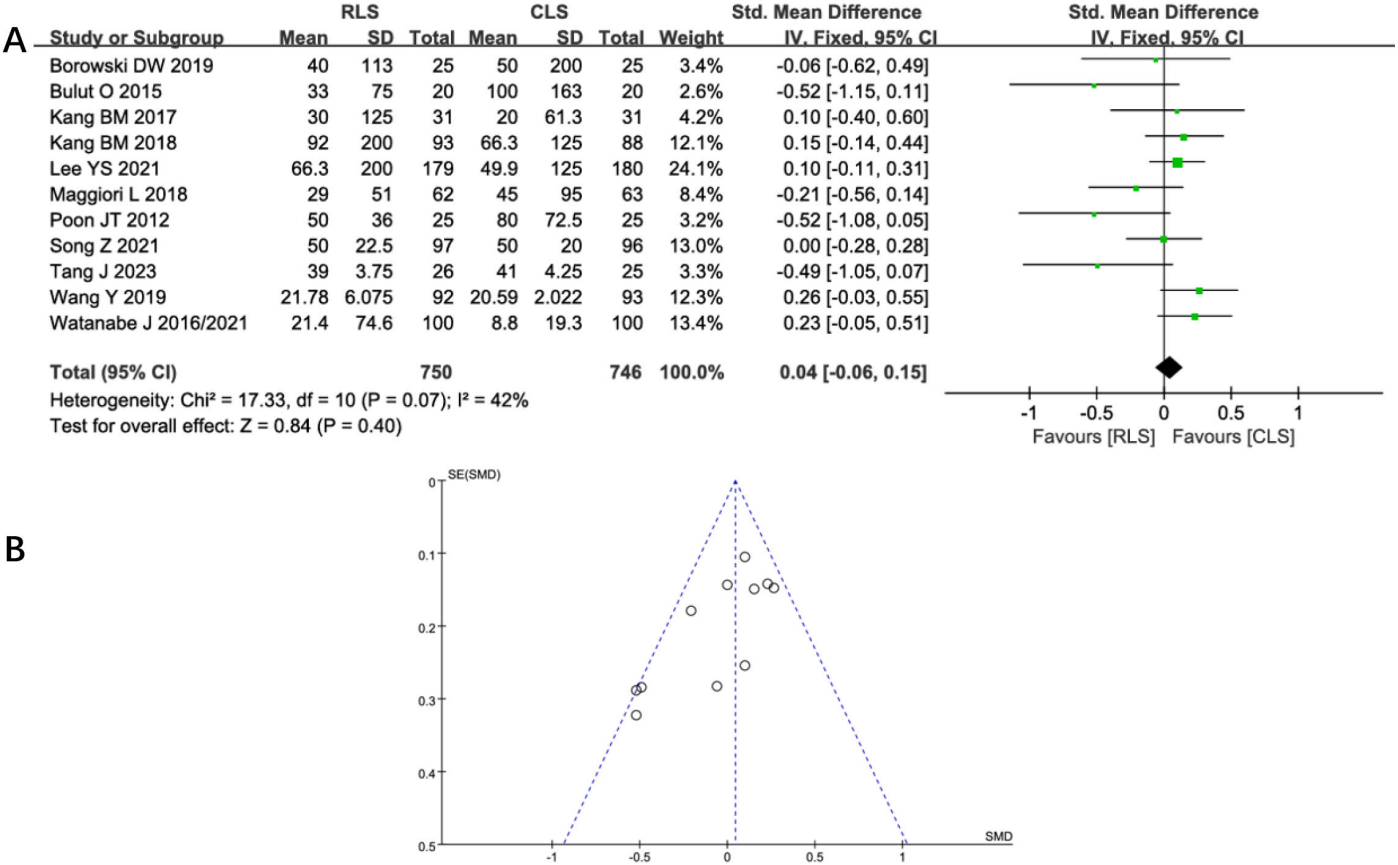
**(A)** Forest plot of intraoperative blood loss comparing RLS and CLS. **(B)** Funnel plot of intraoperative blood loss comparing RLS and CLS. RLS, reduced-port laparoscopic surgery; CLS, conventional laparoscopic surgery.

Twelve studies evaluated operative time, demonstrating no statistically significant difference between RLS and CLS (SMD: 0.29; 95%CI: -0.07 to 0.64; P = 0.11; I² = 90%; **Figure 4**). Significant heterogeneity was observed, prompting sensitivity analysis using the leave-one-out method. Exclusion of studies by Poon et al. (11) and Tang J et al. (21), identified as primary sources of heterogeneity, substantially reduced heterogeneity without altering the overall conclusion (SMD: -0.02; 95% CI: -0.12 to 0.08; P = 0.71, I² = 38%). Further investigation revealed pronounced discrepancies in operative time between RLS and CLS within these two studies, suggesting methodological or population-specific variations as potential contributors to initial heterogeneity. Analysis of intraoperative blood loss across 11 studies similarly showed no significant intergroup difference (SMD: 0.04; 95% CI: -0.06 to 0.15; P = 0.40; I² = 42%; **Figure 5A**), with funnel plot symmetry indicating minimal publication bias (**Figure 5B**). These findings collectively suggest comparable intraoperative efficiency between RLS and CLS approaches.

#### Intraoperative and postoperative complications

**Figure 6:**
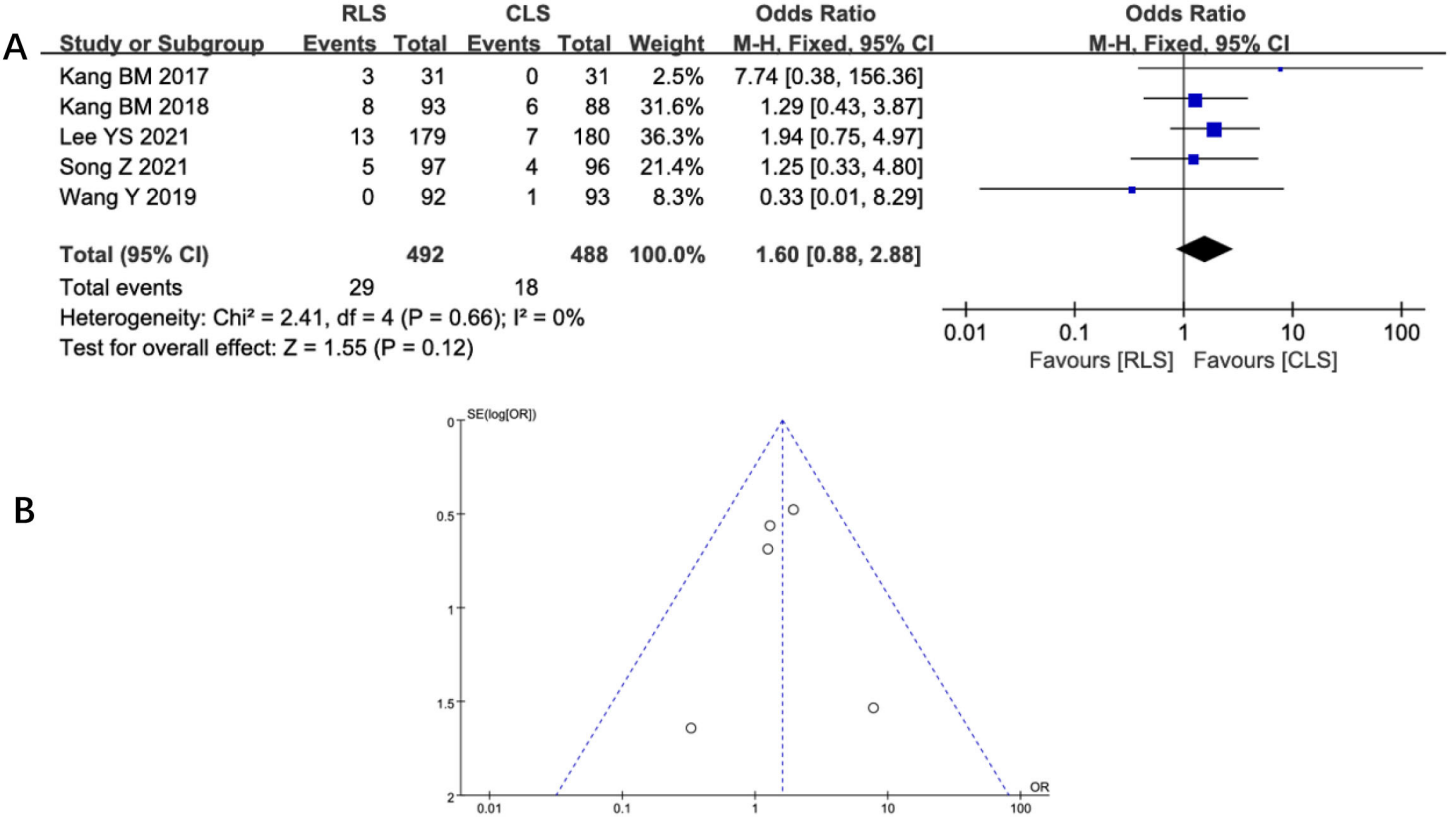
**(A)** Forest plot of intraoperative complications comparing RLS and CLS. **(B)** Funnel plot of intraoperative complications comparing RLS and CLS. RLS, reduced-port laparoscopic surgery; CLS, conventional laparoscopic surgery.

**Figure 7:**
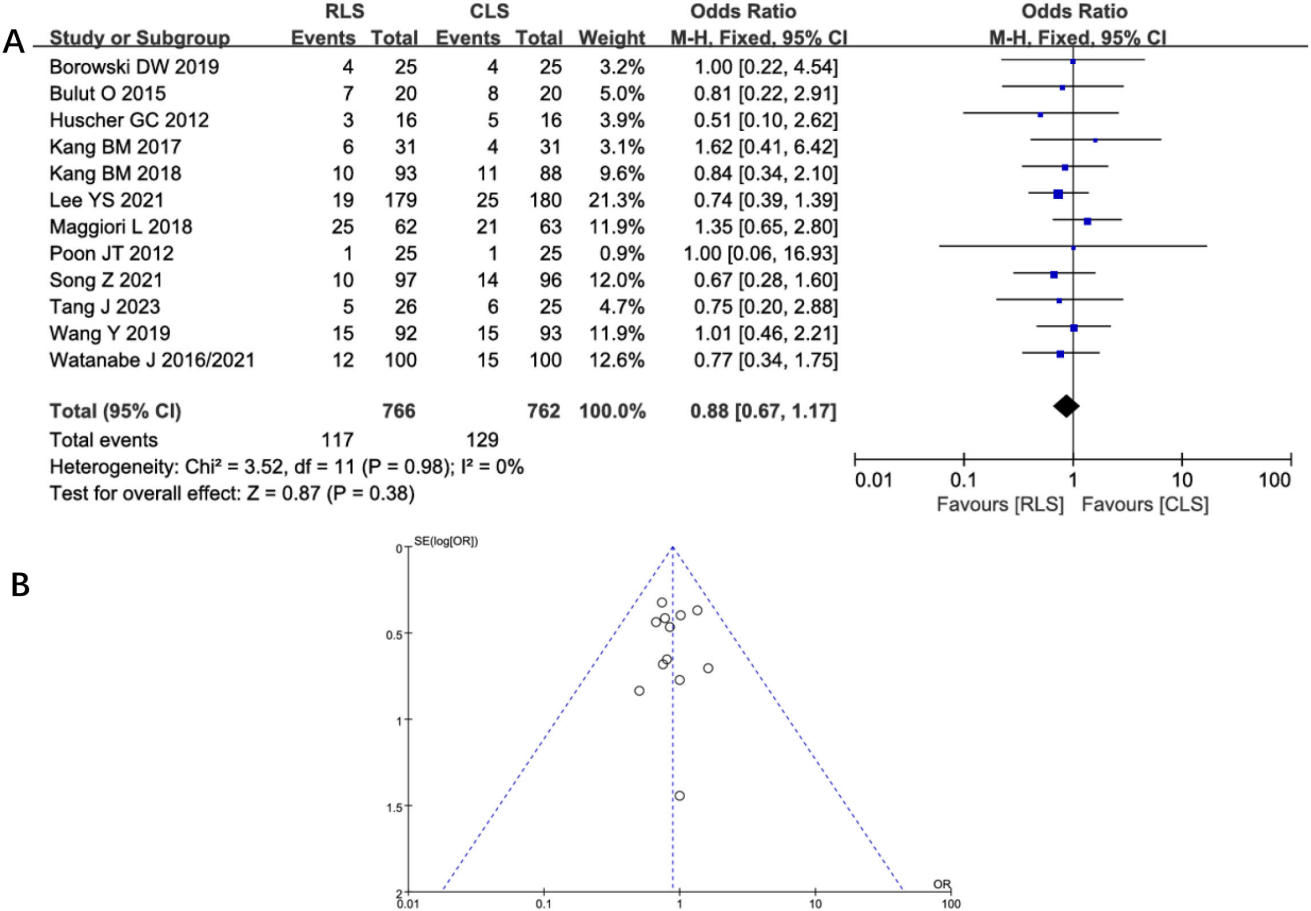
**(A)** Forest plot of postoperative complications comparing RLS and CLS. **(B)** Funnel plot of postoperative complications comparing RLS and CLS. RLS, reduced-port laparoscopic surgery; CLS, conventional laparoscopic surgery.

Five studies assessed intraoperative complications, revealing no statistically significant difference between RLS and CLS (OR: 1.6; 95% CI: 0.88 to 2.88; P = 0.12; I² = 0%; **Figure 6A**). Funnel plot analysis demonstrated symmetrical distribution of studies (**Figure 6B**), suggesting minimal publication bias. Similarly, analysis of postoperative complications across 12 studies showed comparable outcomes between groups (OR: 0.88; 95% CI: 0.67 to 1.17; P = 0.38; I² = 0%; **Figure 7A**), with funnel plot symmetry further supporting the absence of significant bias (**Figure 7B**). The negligible heterogeneity (I² = 0% for both outcomes) underscores methodological consistency across included trials. These findings collectively indicate no clinically meaningful disparity in complication profiles between RLS and CLS approaches.

### Secondary outcomes

#### Analysis of postoperative complication severity

**Figure 8:**
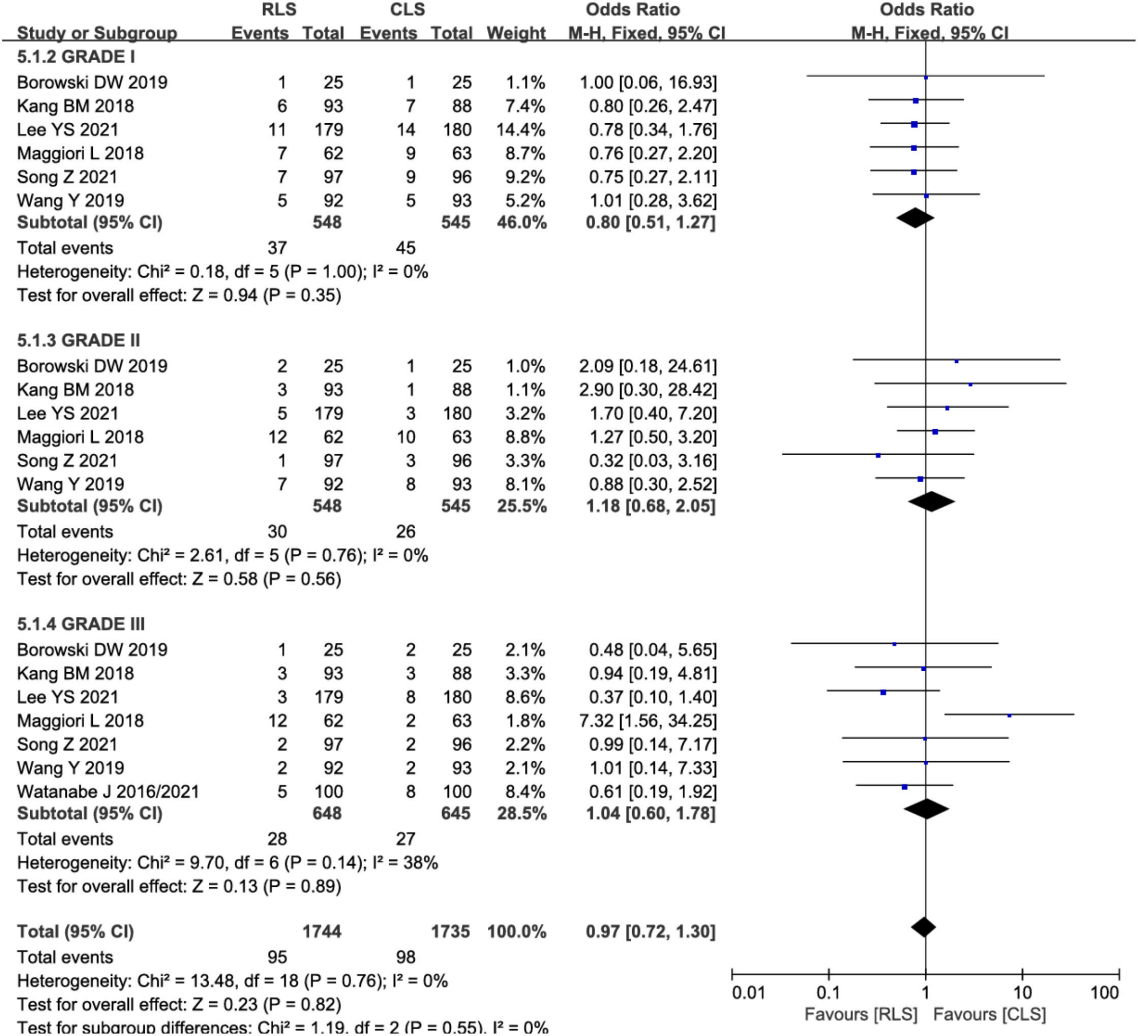
Forest plot of postoperative complication severity comparing RLS and CLS. RLS, reduced-port laparoscopic surgery; CLS, conventional laparoscopic surgery.

Subgroup analyses stratified by Clavien-Dindo classification grades were performed to evaluate complication severity. Six studies reported Grade I-II complications, while seven studies assessed Grade III complications. Pooled analysis demonstrated no significant differences in complication severity between RLS and CLS (OR: 0.97; 95% CI: 0.72–1.30; P = 0.55; I² = 0%; **Figure 8**). The absence of heterogeneity indicates high consistency across studies. Funnel plot symmetry further supported the reliability of these findings, suggesting no substantial publication bias. These results reinforce the comparable safety profiles of RLS and CLS across all severity grades of postoperative complications.

#### Resection margins and lymph nodes harvested

**Figure 9:**
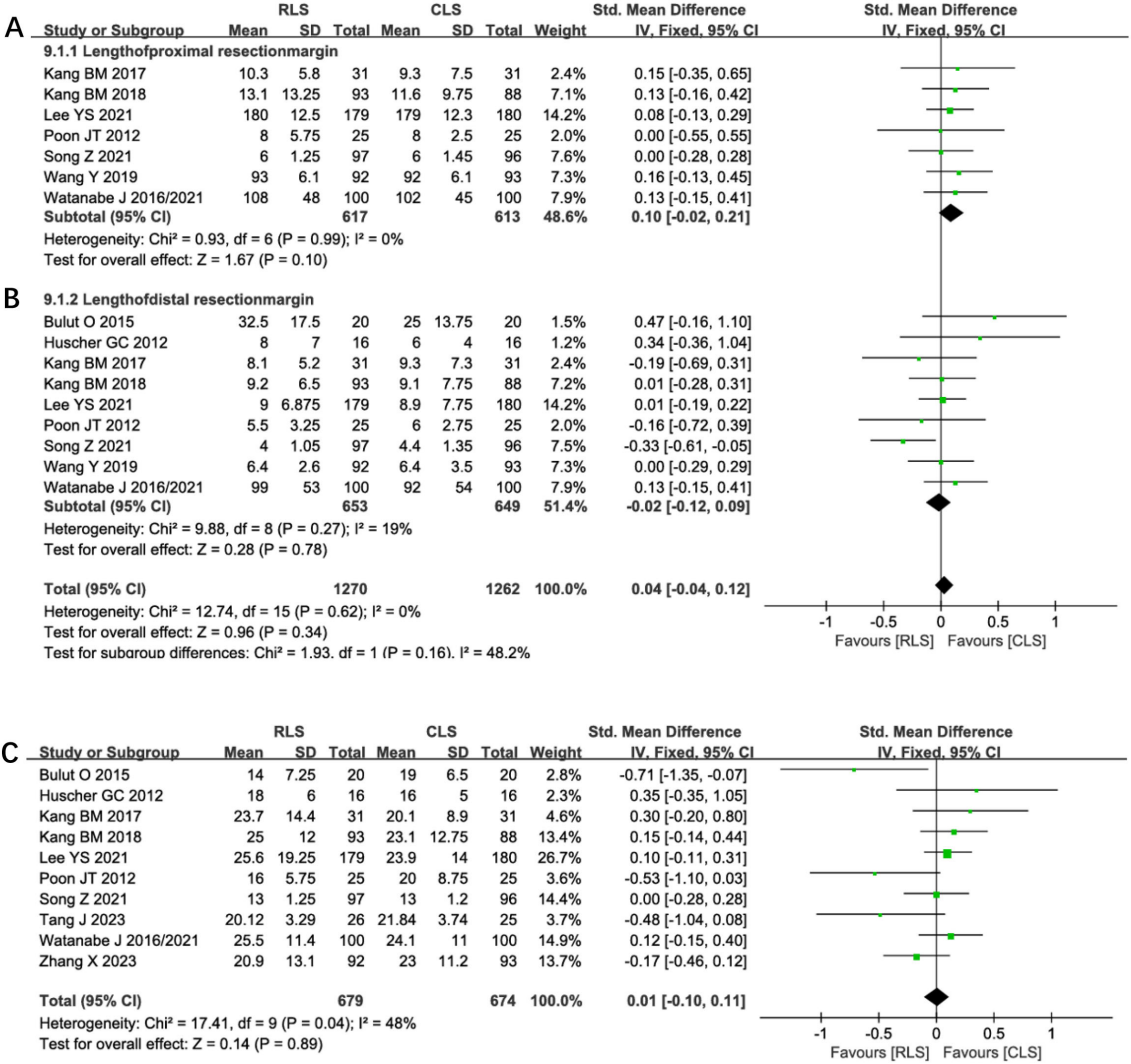
Forest plot of resection margins **(A)** Proximal, **(B)** Distal, **(C)** Lymph nodes harvested comparing RLS and CLS. RLS, reduced-port laparoscopic surgery; CLS, conventional laparoscopic surgery.

Pooled analysis of 7 studies demonstrated no significant difference in proximal resection margins between RLS and CLS (SMD: 0.10; 95% CI: -0.02 to 0.21; P = 0.10; I² = 0%; **Figure 9A**). Similarly, analysis of distal resection margins across 9 studies revealed comparable outcomes (SMD: -0.02; 95% CI: -0.12 to 0.09; P = 0.78; I² = 19%; **Figure 9B**). Evaluation of lymph node Harvested in 11 studies showed no intergroup disparity (SMD: 0.01; 95% CI: -0.10 to 0.11; P = 0.89; I² = 48%; **Figure 9C**). The low heterogeneity (I² ≤48%) across these oncological parameters supports the consistency of surgical quality between techniques, with funnel plots confirming symmetrical study distribution. These findings collectively indicate equivalent oncological adequacy in margin clearance and lymph node dissection for both surgical approaches.

#### Postoperative recovery outcomes

**Figure 10:**
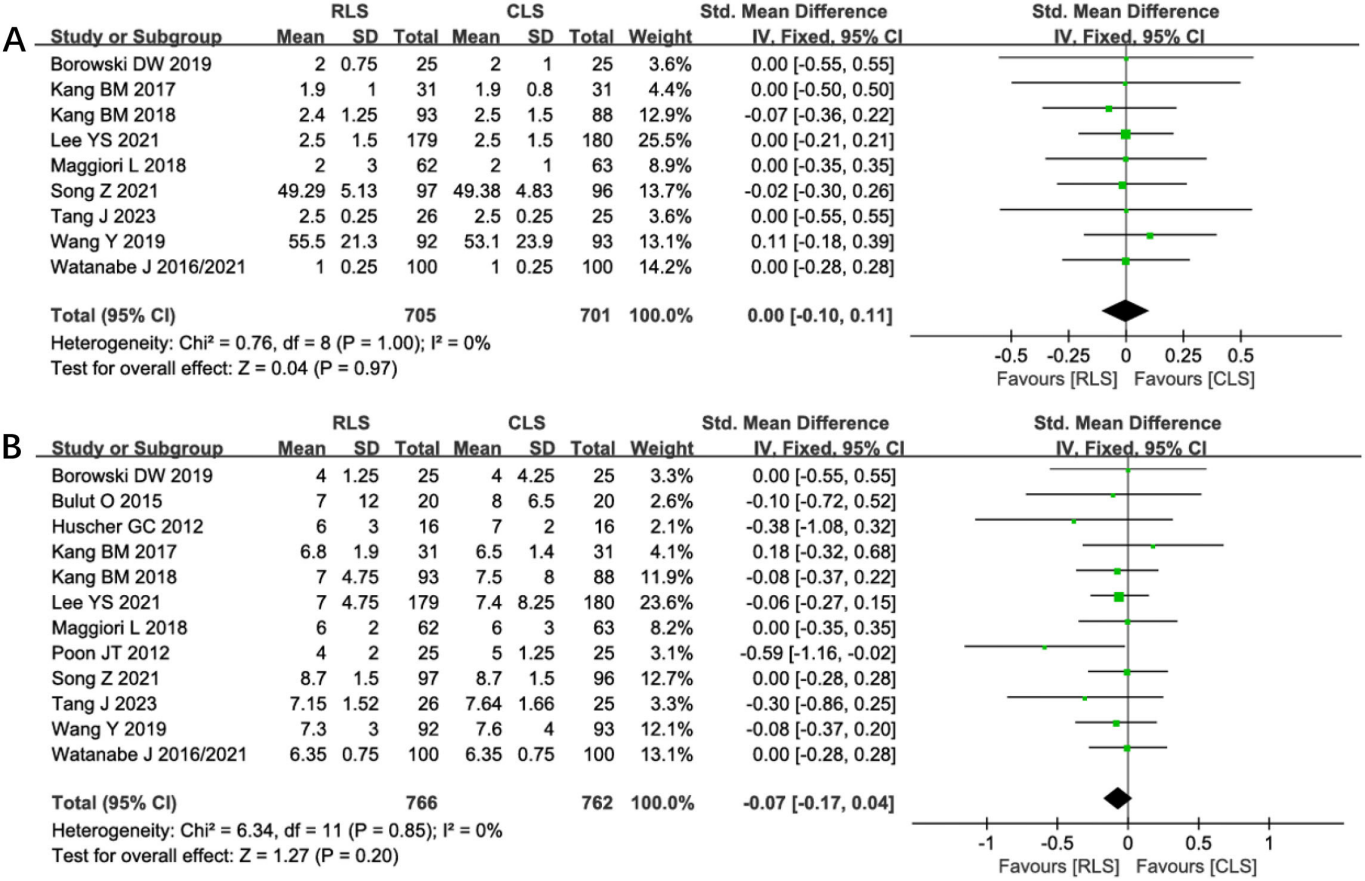
Forest plot of the time to first postoperative flatus and postoperative hospital stay comparing RLS and CLS **(A)** The time to first postoperative flatus; **(B)** postoperative hospital stay. RLS, reduced-port laparoscopic surgery; CLS, conventional laparoscopic surgery.

Nine studies evaluated the time to first postoperative flatus, demonstrating no significant difference between RLS and CLS (SMD: 0.00; 95% CI: -0.10 to 0.11; P = 0.97; I² = 0%; **Figure 10A**). Similarly, analysis of postoperative hospital stay across 12 studies revealed comparable durations between groups (SMD: -0.07; 95% CI: -0.17 to 0.04; P = 0.20; I² = 0%; **Figure 10B**). The absence of heterogeneity indicates consistent findings across all included trials, with funnel plots confirming symmetrical study distributions. These results suggest equivalent postoperative recovery trajectories in gastrointestinal function restoration and hospitalization requirements for RLS and CLS approaches.

#### Incision length, conversion rates, and anastomotic leakage

**Figure 11:**
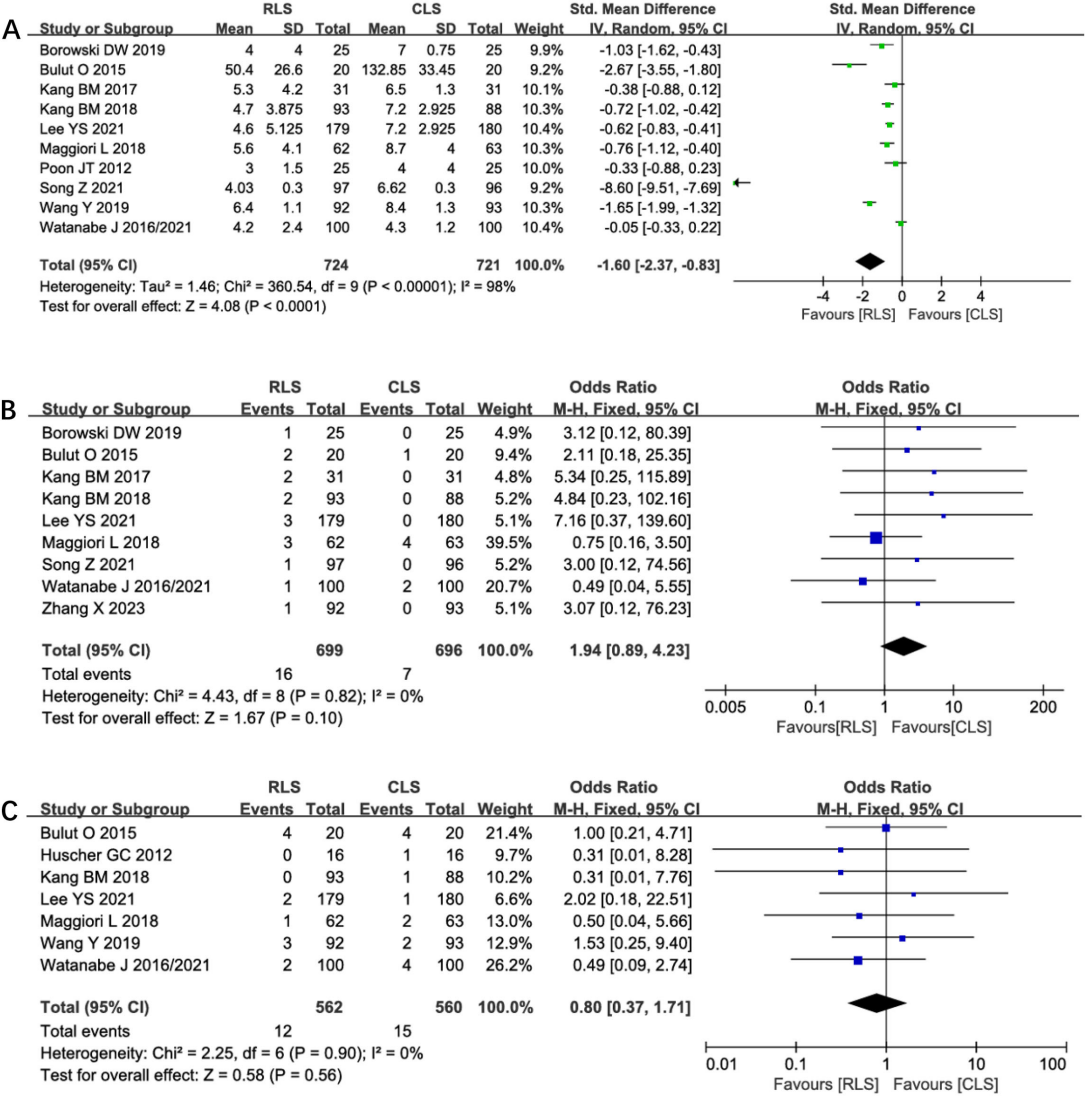
Forest plot of incision length, conversion rates, anastomotic leakage comparing RLS and CLS **(A)** Incision length; **(B)** Conversion rates; **(C)** Anastomotic leakage. RLS, Reduced-port laparoscopic surgery; CLS, conventional laparoscopic surgery.

Analysis of incision length across 10 studies revealed a statistically significant reduction in total incision length favoring RLS over CLS (SMD: -1.60; 95% CI: -2.37 to -0.83; P < 0.0001; I² = 98%; **Figure 11A**). Substantial heterogeneity prompted sensitivity analysis, which identified four outlier studies contributing to heterogeneity. Exclusion of these studies maintained statistical significance while eliminating heterogeneity (SMD: -0.65; 95% CI: -0.79 to -0.51; P < 0.00001; I² = 0%), suggesting methodological variability in incision measurement as a potential confounder. Nine studies reported conversion rates to open surgery, showing no significant intergroup difference (OR: 1.94; 95% CI: 0.89 to 4.23; P = 0.10; I² = 0%; **Figure 11B**). Similarly, analysis of anastomotic leakage in seven studies demonstrated comparable rates between RLS and CLS (OR: 0.80; 95% CI: 0.37 to 1.71; P = 0.56; I² = 0%; **Figure 11C**). The absence of heterogeneity and symmetrical funnel plots reinforce the reliability of these findings. These results collectively confirm RLS’s cosmetic superiority without compromising procedural feasibility or anastomotic safety.

#### Postoperative pain

**Figure 12:**
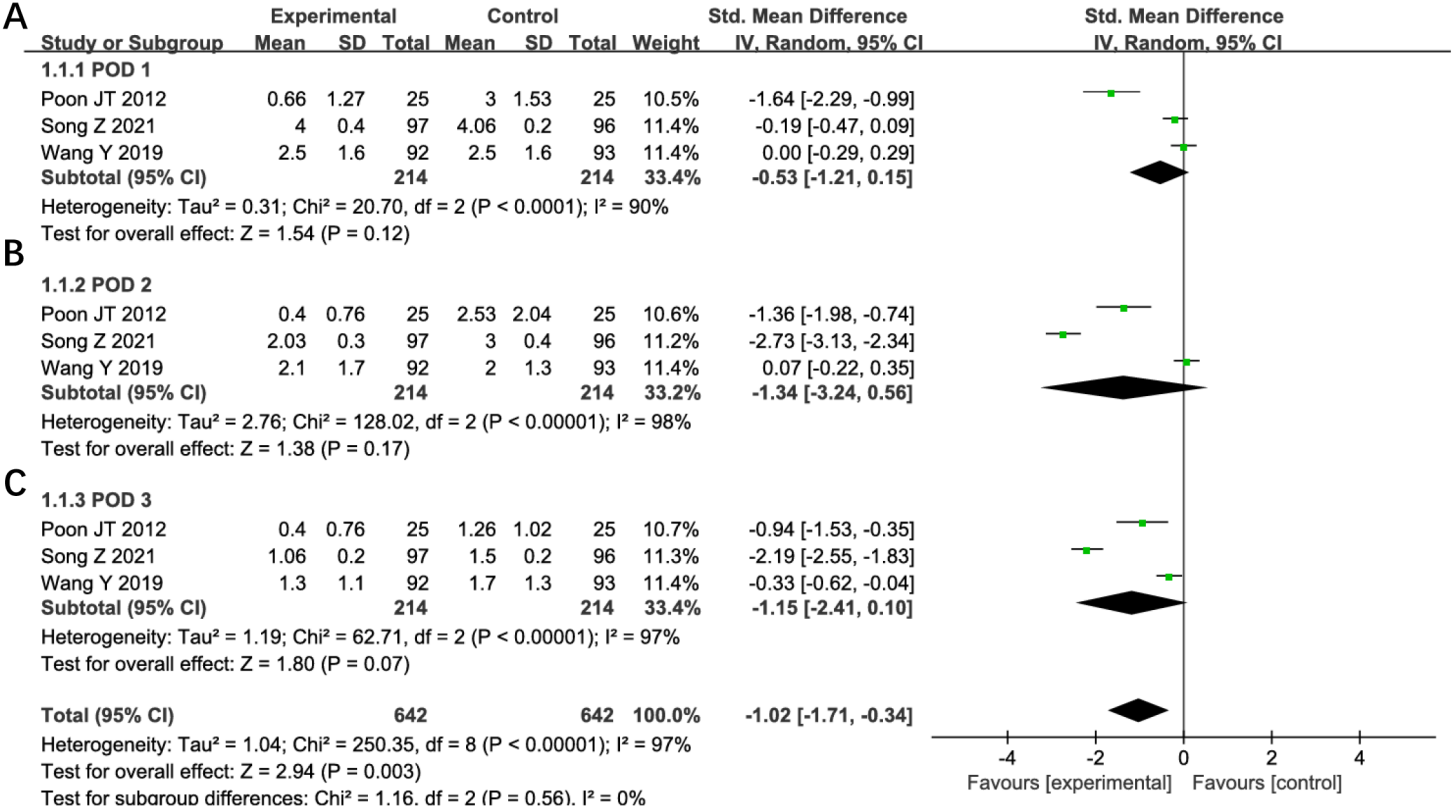
Forest plot of POD1, POD2, POD3 pain comparing RLS and CLS **(A)** POD1, **(B)** POD2, **(C)** POD3. RLS, reduced-port laparoscopic surgery; CLS, conventional laparoscopic surgery; POD, postoperative day.

Five studies evaluated postoperative pain outcomes in this analysis. Two studies were excluded from quantitative synthesis due to reporting only mean pain scores without standard deviations. Meta-analysis of the remaining three studies demonstrated no statistically significant differences in pain scores between groups during the first three postoperative days: day 1 (SMD -0.53, 95% CI -1.21 to 0.15; P=0.12; I²=90%, **Figure 12A**), day 2 (SMD -1.34, 95% CI -3.24 to 0.56; P=0.17; I²=98%, **Figure 12B**), and day 3 (SMD -1.15, 95% CI -2.41 to 0.10; P=0.07; I²=97%, **Figure 12C**). Substantial heterogeneity was observed across all time points. Sensitivity analysis failed to identify specific sources or reduce the heterogeneity, though differences in pain assessment methodologies may represent a potential contributing factor. Given the high degree of between-study variability, these findings regarding postoperative pain should be interpreted with appropriate caution. The numerical trends favoring reduced-port surgery, while not statistically significant, may warrant further investigation in studies with standardized pain evaluation protocols.

### Trial sequential analysis

**Figure 13:**
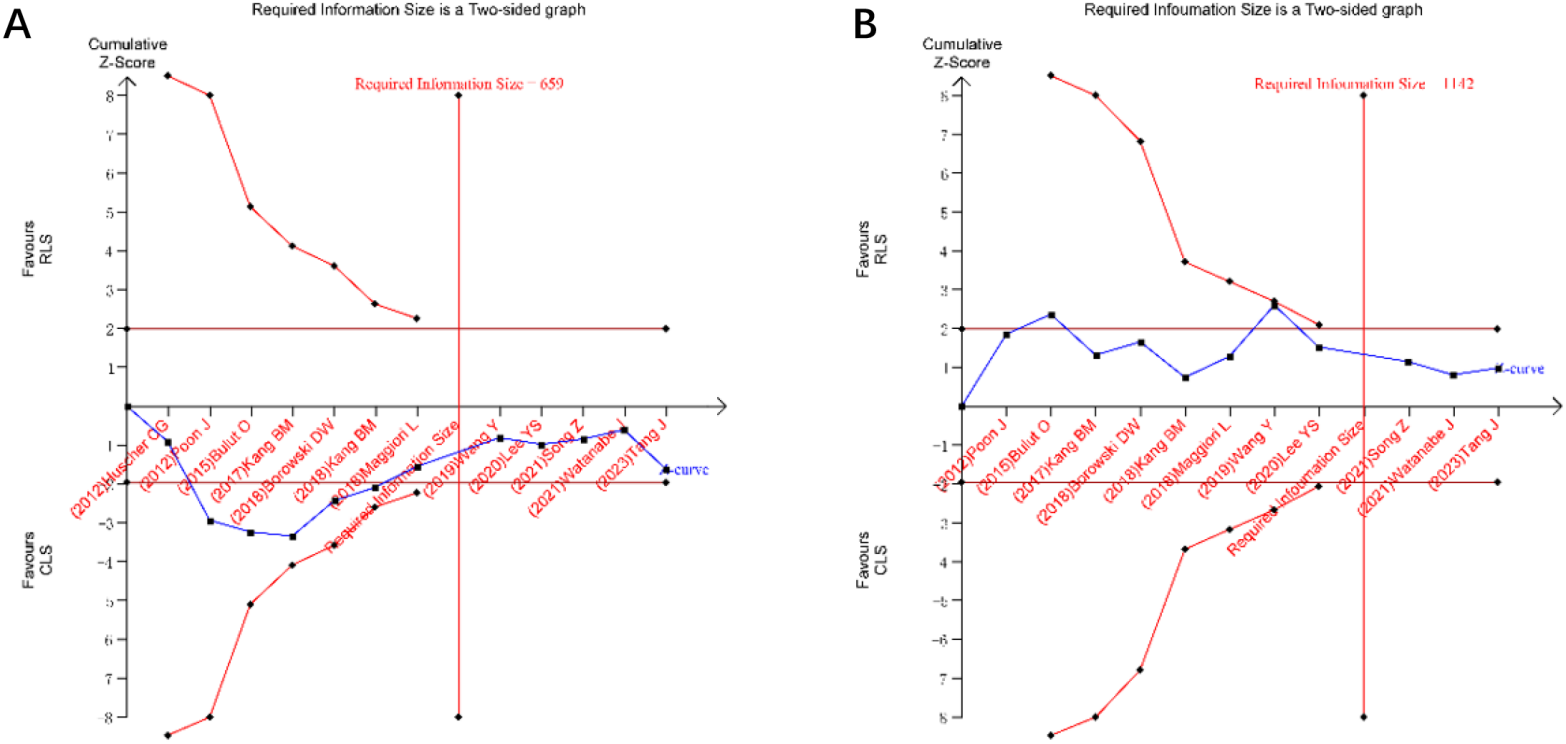
Trial sequential analysis for operative time and intraoperative blood loss **(A)** operative time; **(B)** intraoperative blood loss.

**Figure 14:**
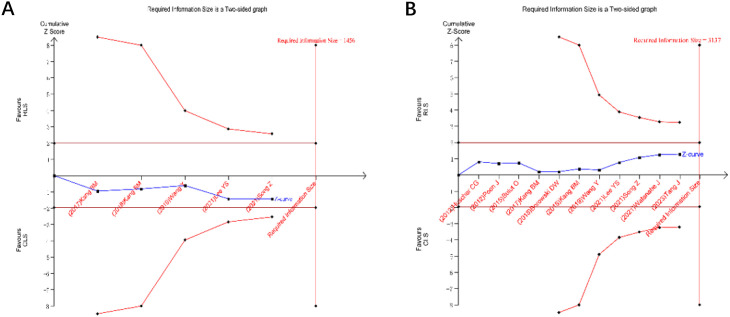
Trial sequential analysis for intraoperative and postoperative complications **(A)** intraoperative postoperative complications; **(B)** postoperative complications.

TSA was performed for primary outcomes using predefined monitoring boundaries (α = 0.05, power = 80%). For operative time and intraoperative blood loss (**Figures 13**), the cumulative Z-curves crossed neither the conventional nor TSA-adjusted significance boundaries, despite surpassing the required information size. This confirms the stability of non-significant differences between RLS and CLS for these outcomes. Analysis of intraoperative and postoperative complications (**Figures 14**) revealed that the cumulative Z-curves remained within futility boundaries without reaching RIS thresholds. While current evidence suggests no statistically significant intergroup differences, the insufficient cumulative sample size indicates that additional RCTs are required to conclusively exclude clinically meaningful disparities.

## Discussion

Laparoscopic colorectal resection stands as the most common surgical approach in gastrointestinal surgery. In 1991, Dr. Jacob et al. (22) from the United States reported the first case of laparoscopic colectomy for colorectal cancer, marking the beginning of the rapid development of minimally invasive techniques centered on laparoscopy in colorectal surgery. To date, the technique has progressively evolved to fully robot-assisted laparoscopic colectomy. Fully robotic laparoscopic colorectal surgery has been demonstrated as safe and effective (23, 24), without leading to longer operative times or increased blood loss (25). Building on conventional laparoscopy, RLS such as SILS and SILS+1 have gained attention, particularly SILS, which has been widely adopted in fields including hepatobiliary (26), gynecology (27, 28), and urology (29), and has further evolved into robotic SILS (30–34). In contrast, SILS+1 procedures remain less frequently reported (35, 36). Single-incision laparoscopic colorectal resection was first described in 2018 (37). Compared to conventional laparoscopy, it offers reduced postoperative pain, superior cosmetic outcomes, and non-inferior long-term oncological results, including 3-year DFS and 5-year OS (5, 20). However, most supporting studies are retrospective (38, 39), limiting their evidence quality. Despite favorable outcomes, the technical complexity and steep learning curve of SILS and SILS+1, particularly for SILS, have hindered widespread adoption (40). Short-term outcomes remain controversial. A meta-analysis by ElSherbiney M et al. (6) found no differences in operative time or overall complications between SILS and CLS but reported higher conversion rates to open surgery. Conversely, Li FH et al. (7) concluded that SILS lacks comprehensive advantages over conventional laparoscopy and may be associated with higher complication rates. To address these inconsistencies, our meta-analysis incorporated updated RCTs evaluating both SILS and SILS+1 techniques, collectively termed RLS. Pooled results demonstrated that RLS achieves shorter incisions compared to CLS but shows no significant advantages in resection margins, lymph node yield, time to first flatus, hospital stay, conversion rates, or anastomotic leakage. For patients prioritizing cosmesis, RLS may serve as an alternative, provided it is performed by experienced surgeons.

While our study primarily focused on short-term surgical outcomes, long-term oncological results warrant equal attention. Among the included studies, two publications reported long-term follow-up data. Huscher CG et al. (4) conducted a median 22-month follow-up, identifying 2 cases of tumor recurrence; however, the study did not specify which surgical group experienced recurrence. Notably, neither group exhibited port-site metastases. Bulut O et al. (12) provided extended follow-up data over 15 months, documenting 2 cases of recurrence and 1 late anastomotic leakage in the CLS group, whereas the RLS group had 1 case of stoma prolapse. Regarding incisional hernia risk, only one study systematically compared both groups. Longitudinal data spanning 36 months postoperatively revealed 17 total cases of incisional hernia, with incidence rates of 9.6% in the CLS group versus 8.3% in the RLS group. This non-significant difference may be attributed to the fact that SILS/SILS+1 techniques primarily reduce the number of trocars without modifying specimen extraction incision length – a key determinant of hernia development. Multivariate analysis revealed that the occurrence of incisional hernias was significantly associated with body mass index, history of umbilical hernia, and umbilical incision length (20).

Trial sequential analysis, a method to estimate sample size and enhance result robustness by minimizing repetitive hypothesis-testing risks from new studies (41, 42), was applied to key outcomes. TSA confirmed the stability of non-significant differences in operative time and blood loss between RLS and CLS, as cumulative Z-curves surpassed the required information size without crossing significance thresholds. However, the absence of differences in intraoperative and postoperative complications requires validation through additional RCTs.

### Study limitations

First, the forest plot analysis comparing incision length revealed significant bias among studies, and the exclusion of numerous studies during sensitivity analysis may have compromised the reliability of the conclusions. Second, substantial heterogeneity was observed in postoperative pain outcomes between the two groups, which further affects the robustness of the findings. Additionally, the inability to perform quantitative analysis of long-term oncological outcomes precludes assessment of whether the technical challenges associated with RLS impact patient survival. Finally, the inclusion of both single-incision and single-incision plus one laparoscopic colorectal resection techniques in the pooled analysis may have influenced result validity.

### Conclusion

RLS is safe and effective for colorectal cancer patients. It may be considered for those with high cosmetic demands but should be performed by surgeons proficient in advanced laparoscopic techniques.
